# Clinical characteristics, treatment, and long-term outcome of patients with brain metastases from thyroid cancer

**DOI:** 10.1007/s10585-023-10208-8

**Published:** 2023-05-23

**Authors:** Ladislaia Wolff, Ariane Steindl, Petar Popov, Karin Dieckmann, Brigitte Gatterbauer, Georg Widhalm, Anna Sophie Berghoff, Matthias Preusser, Markus Raderer, Barbara Kiesewetter

**Affiliations:** 1 Department of Medicine I, Division of Oncology, Medical University of Vienna, Vienna, Austria; 2 Department of Biomedical Imaging and Image-guided Therapy, Division of Nuclear Medicine, Medical University of Vienna, Vienna, Austria; 3 Department of Neurosurgery, Medical University of Vienna, Vienna, Austria

**Keywords:** Papillary, Follicular, Medullary, Anaplastic thyroid cancer, Brain metastases, Real world data

## Abstract

Brain metastases (BM) in patients with thyroid cancer (TC) are rare with an incidence of 1% for papillary and follicular, 3% for medullary and up to 10% for anaplastic TC (PTC, FTC, MTC and ATC). Little is known about the characteristics and management of BM from TC. Thus, we retrospectively analyzed patients with histologically verified TC and radiologically verified BM identified from the Vienna Brain Metastasis Registry. A total of 20/6074 patients included in the database since 1986 had BM from TC and 13/20 were female. Ten patients had FTC, 8 PTC, one MTC and one ATC. The median age at diagnosis of BM was 68 years. All but one had symptomatic BM and 13/20 patients had a singular BM. Synchronous BM at primary diagnosis were found in 6 patients, while the median time to BM diagnosis was 13 years for PTC (range 1.9–24), 4 years for FTC (range 2.1–41) and 22 years for the MTC patient. The overall survival from diagnosis of BM was 13 months for PTC (range 1.8–57), 26 months for FTC (range 3.9–188), 12 years for the MTC and 3 months for the ATC patient. In conclusion, development of BM from TC is exceedingly rare and the most common presentation is a symptomatic single lesion. While BM generally constitute a poor prognostic factor, individual patients experience long-term survival following local therapy.

## Introduction

Thyroid cancer (TC) is a rare type of cancer with an incidence of 8/100.000 people in Austria per year affecting mostly women with a female/male ration of 2:1 [1]. Clinical characteristics, prognosis and treatment of TC depend on the histological subtype. Well differentiated papillary TC (PTC) represent about 75% of all thyroid malignancies and has the best prognosis with a median 10-year overall survival (OS) of > 90% [5] while the prognosis is less favorable in less differentiated TC, medullary (MTC, 10-year OS 75%) and drastically worse in anaplastic TC (ATC) with a median OS in the range of only a few weeks to months [6].

The therapeutic algorithm for TC is dependent on the histological subtype and stage, ranging from potentially curative surgery in localized TC to radioactive-iodine (RAI) therapy for differentiated TC (DTC), (multi)tyrosine kinase inhibitors (TKI) for RAI-refractory DTC and metastatic MTC and radio/chemotherapy for ATC [7]. Recently, molecular driven therapies have also been evaluated and include NTRK inhibitors for TC with *NTRK* fusions and RET inhibitors for *RET*-activated thyroid malignancies [9]. Also, *BRAF*-targeted therapies, including RAI-redifferentiation strategies in DTC are of interest and are currently explored [10–13].

Despite an increasing number of targeted therapies being approved for systemic therapy of advanced TC, the presence of distant metastases is a relevant event in the clinical course associated with a drastically worse prognosis. Whereas synchronous lymph node involvement is a frequent finding in localized TC, distant metastases are present in less than 5% of DTC, up to 15% in MTC and 50% in ATC at primary diagnosis [7, 14]. During the course of the disease, approximately 15% of patients with DTC, 25% with MTC and > 90% with ATC develop distant metastases [15, 16]. Regarding localization of distant metastases, organ metastases most commonly occur in the lungs (> 70%) followed by bone manifestations (20%) [8, 17].

According to the literature, brain metastases (BM) constitute a rare finding in TC with an incidence of only 1% in DTC, 4% in MTC and less than 10% even in the aggressive ATC cohort. As a consequence, no current guidelines for the management of BM from TC exist [19] and data on patients with BM form TC are scarce. While it has been documented that the presence of BM correlates with a worse prognosis compared to only distant extracranial metastases, in fact very few patient series are available investigating the onset, presentation and clinical course of patients with BM and their specific management (see Table 1) [4, 8, 9, 20–26].

**Table 1:** Baseline characteristics

Patient characteristics	at diagnosis of TC	at diagnosis of BM
Sex female/male	13/7	
Histology: PTC/FTC/ATC/MTC	8/10/1/1	
**Age**		
Papillary	56 (46–73)	72 (46–75)
Follicular	54 (21–75)	62 (45–75)
Anaplastic	66	66
Medullary	50	71
**ECOG-PS**		
0	12 (60%)	2 (10%)
1	8 (40%)	16 (65%)
2	0	2 (10%)
**Localisation of metastases**	**N = 8**	** N = 20**
Lung and bones	2	0
Lung, bones and brain	6	19
Lung, bones, adrenal gland and brain	0	1
**Number of brain metastases**	**N = 6**	** N = 20**
1	4	13
2	1	4
3	0	2
> 3	1	1
**Localization of brain metastases**	**N = 6**	** N = 20**
Left hemisphere	4	8
Right hemisphere	1	6
Both hemispheres	1	4
Cerebellum	0	2
**Symptoms of brain metastases**	**N = 6**	** N = 20**
Neurological deficits	5	11
Increased intracranial pressure	1	5
Seizures	0	3
Asymptomatic BM	0	1
**Upfront treatment of brain metastases**		
Resection		7
Stereotactic radiosurgery		4
Resection and SRS		4
WBRT		3
BSC		2

*ATC, anaplastic thyroid cancer; BM, brain metastases; BSC, best supportive care; ECOG-PS, eastern cooperative oncology group - performance status; FTC, follicular thyroid cancer, MTC, medullary thyroid cancer; N, number; PTC, papillary thyroid cancer; PDTC, poorly-differentiated thyroid cancer; SRS, stereotactic radiotherapy; TC, thyroid cancer, WBRT, whole brain radio therapy*

The Medical University of Vienna is a tertiary referral center for patients with TC and management is subject to multidisciplinary teams of specialized medical oncologists, endocrine surgeons, endocrinologists, and nuclear medicine physicians, thus spanning the entire spectrum and longitudinal course from initial diagnosis to metastatic disease. A comprehensive database, the Vienna Brain Metastases Registry, on patients diagnosed with BM between 1986 and 2020 includes not only pathological characteristics but also BM-associated clinical data such as metric information and neurological symptom burden [27]. This offers the unique opportunity to thoroughly analyze clinical characteristics, treatment, and long-term outcome of patients with BM from TC treated at the Medical University of Vienna. Within the current analysis, we aim to further characterize the course of this rare patient cohort in order to potentially optimize management and therapeutic strategies.

## Methods

### Patient selection

The current analysis is a retrospective review of patients with a histologically verified diagnosis of TC and documented BM treated at the Medical University of Vienna between 1986 and 2020. Patients with TC and BM were identified from the Vienna Brain Metastasis Registry, a database established at the Division of Oncology, Medical University of Vienna, including patients with cerebral metastases since 1986 [28]. General clinical data as well as BM-specific data were retrospectively extracted from routine medical records and anonymized for further analysis. This investigation has been approved by the local ethical board of the Medical University of Vienna (EK No 1692/2022).

### Baseline characteristics

Baseline characteristics and the clinical course of patients were retrieved from electronic medical records including sex, age, Eastern Cooperative Oncology Group performance status (ECOG-PS) at diagnosis of TC and BM, histological subtype, staging at primary diagnosis, time to development of distant metastases and BM, progression and OS. Distant metastases were defined as extracranial metastases other than BM. The histological subtype was documented and included PTC, FTC, MTC and ATC. Furthermore, treatment modalities and response to therapies (surgical resection, number and cumulative dose of RAI therapies, systemic radio-/chemo-/targeted therapies), the clinical course and survival outcomes were assessed, i.e., progression-free survival (PFS), OS and cause of death, if applicable.

### BM specific characteristics

The diagnosis of BM was established with computed tomography, magnetic resonance imaging, whole-body I-131 scan or 18 F-FDG-positron emission computed tomography. In addition, histological assessment was performed in patients undergoing resection of BM. Documented treatment options included surgical intervention, stereotactic radiosurgery (SRS), whole-brain radiation therapy (WBRT), external beam radiation therapy (EBRT) or best supportive care (BSC). For this analysis, we have documented and grouped symptoms caused by BM as follows: (1) *neurological deficits*: including aphasia, sensorimotor dysfunctions, vertigo, impaired vision and cognitive impairment; (2) *increased intracranial pressure*: emesis and headache and (3) *epileptic seizures* including focal and generalized seizures. We have furthermore assessed the number, size (in cm) and localization of intracranial lesions. The number of BM were grouped into one, two or three and more than three lesions according to the classification in the BM Registry.

### Statistical analysis

TC diagnosis was determined as the date of histological verification, usually after thyroidectomy. Diagnosis of TC and BM was defined as “synchronous” when BM were diagnosed three months before or after diagnosis of TC. Disease progression was determined as the radiological diagnosis of a local recurrence in the curative setting, i.e. total surgical removal of all tumor or as a progression / new lesions of distant metastases or BM in the palliative, i.e. non-resectable setting. The time from TC diagnosis to local, distant, or intracranial progression was assessed in months. PFS was defined as the time from initiation of a respective therapy to progression or death. OS was calculated twice: starting from TC- and from BM-diagnosis until death or last follow-up. The follow-up time was measured from the date of first diagnosis to death or last follow-up. SPSS version 26 (SPSS Inc., Chicago, USA) was used for statistical calculations. Metric variables were presented with medians and ranges (minimum/maximum), categorical variables with absolute and relative (percentage) frequencies. Association of binary variables was assessed using cross tabs and the Fisher’s exact test. Survival times (PFS, OS) were estimated using the Kaplan Meier method and presented with 95% confidence intervals (CI). Comparisons of survival groups were performed with the log-rank test. P-values less than 0.05 (two-sided) were accepted as statistically significant. No adjustment for multiple testing was applied based on the hypothesis-generating and exploratory design of the present study.

## Results

### Baseline characteristics at primary diagnosis

The Vienna Brain Metastasis Registry consisted of 6074 patients at the cut-off date, December 31st, 2021. In total, 20 (0.3%) patients had a histologically verified diagnosis of TC and radiologically verified BM and were thus included in this analysis, see Table 3. In 9 patients, also histological verification of BM could be established after resection. Thirteen patients out of 20 (65%) were female and 7/20 (35%) male; 18/20 presented with DTC, including 8 patients with PTC and 10 with FTC. One patient had MTC and one ATC. The median follow-up time calculated from diagnosis was 78 months (95% CI, 64–93 months). The median age at diagnosis of TC was 56 years (range 21–75 years). There was no relevant difference in median age at diagnosis between FTC and PTC (54 vs. 56 years, respectively). The two patients presenting with MTC and ATC were 50 and 66 years, respectively. ECOG-PS at initial diagnosis was good in all patients, with 12 (60%) patients rated as ECOG-PS 0, and 8 (40%) presenting with an ECOG-PS of 1.

**Table 2:** Individual clinical findings of patients with BM from TC

Pat.ID	Sex/histology	Year of TC/DM/BM-diagnosis/death	Age (TC)	ECOG-PS TC/BM	TNM stage	Localization of DM (BM)	Months from TC to DM/to BM	Upfront TC therapy (n. RAIT)/cum. RAIT dose (GBq)	Upfront/2L BM treatment*	BM number/ localisation/ size/ symptoms**	Systemic therapy	Last status (cause)
1	F/PTC	1981/2000/2005/2005	51	0/1	T2 N0 M0	lung, bones	232/288	TE, RAIT (7x)/37,67	SRS/0	2/both/1,5/def.	NA	death (cerebral)
2	M/PTC	1984/1994/2003/2003	53	1/1	T4 N1 M0	lung, bones	115/227	TE, RAIT (3x)/16,65	OP + SRS/0	3/right/5/def.	NA	death (cerebral)
3	M/PTC	1986/1996/1998/2001	61	0/1	T4 N1 M0	lung, bones	119/145	TE, RAIT (4x)/NA	OP + SRS/0	3/left/3/def.	NA	death (extracerebral)
4	F/PTC	2007/2020/2020/alive	46	0/1	T3 N0 M0	lung, bones	160/161	TE, RAIT (1x)/NA	OP/0	1/left/NA/pres.	Lenvatinib	AWD
5	M/PTC	2015/2017/2017/alive	58	0/1	T3 N1 M0	lung, bones	22/23	TE, RAIT (2x)/7,77	OP/0	1/right/0,6/def.	Lenvatinib	AWD
6	F/PTC	2015/2015/2016/2017	70	1/1	T3 N0 M1	lung, bones (l-spine)	0/11	TE, RAIT (2x)/3,73	OP + SRS/WBRT	3/both/1,7/epi.	NA	death (cerebral)
7	M/PTC	2007/2007/2007/2008	46	1/1	T4 N1 M1	lung, bones (sacrum)	0/0	TE, RAIT (1x)/55	WBRT/SRS	8/both/NA/pres.	NA	death (cerebral)
8	M/PTC	2016/2016/2016/2017	73	1/1	T4 N1 M1	lung, bones (c-spine)	0/0	NA/NA	SRS/0	1/left/1/pres.	NA	death (cerebral)
9	F/FTC	1965/2007/2007/2007	21	0/0	T4 N0 M0	lung, bones, adrenal glands	494/495	TE, RAIT (3x)/20,91	OP/0	1/left/NA/epi.	CHT	death (extracerebral)
10	F/FTC	1992/2001/2001/2017	61	0/0	T4 N0 M0	lung, bones (scapula)	102/102	TE, RAIT (6x)/32,375	OP/SRS	1/left/0,9/def.	NA	death (cerebral)
11	F/FTC	1977/1985/1995/1998	35	0/1	T2 N1 M0	lung, bones	96/227	TE, RAIT (10x)/52,91	OP + SRS/SRS	2/both/4/def.	NA	death (extracerebral)
12	F/FTC	2013/2015/2017/2021	48	0/1	T4 N1 M0	lung, bones (c-spine)	22/47	TE, RAIT (2x)/NA	OP/0	1/cere./NA/def.	Lenvatinib	death (extracerebral)
13	M/FTC	1990/1992/1993/1996	59	0/1	T4 N1 M0	lung, bones	25/36	TE, RAIT (3x)/18,12	OP/OP	1/left/6/def.	NA	death (extracerebral)
14	F/FTC	2002/2002/2002/2008	69	1/2	T1 N0 M1	lung, bones (rib)	0/0	RAIT (7x)/43,83	SRS/SRS	1/left/NA/asy.	NA	death (cerebral)
15	M/FTC	2010/2010/2011/2013	44	1/1	T4 N1 M1	lung, bones (femur)	0/8	RAIT (5x)/NA	BSC/0	1/right/1,9/def.	CHT (temozolomid)	death (extracerebral)
16	F/FTC	1994/1995/1996/1997	69	0/2	T4 N1 M0	lung, bones	17/25	TE, RAIT (9x)/47,36	OP/WBRT	1/left/NA/def.	NA	death (extracerebral)
17	F/FTC	1998/1998/1998/1999	49	1/1	T4 N0 M1	lung, bones (rib)	0/2	NA/NA	BSC/0	1/right/7/def.	NA	death (cerebral)
18	F/FTC	2013/2013/2013/2013	75	1/1	T3 N1 M1	lung, bones (tibia)	0/0	TE/NA	WBRT/0	1/right/6/pres.	NA	death (cerebral)
19	M/ATC	1993/1993/1994/1994	66	0/1	T4 N0 M1	lung, bones	0/6	TE/NA	WBRT/SRS	6/right/NA/epi.	CHT (doxorubicine)	death (cerebral)
20	F/MTC	1984/2003/2005/2017	50	0/1	T3 N0 M0	lung, bones (c-spine)	233/257	TE/NA	SRS/0	1/cere./0,3/pres.	CHT	death (extracerebral)

*AWD, alive with disease; asy, asymptomatic; ATC, anaplastic thyroid cancer; BM, brain metastases; BM size, biggest diameter in cm; BSC, best supportive care; cere, cerebellum; CHT, chemotherapy; cum, cummulative; def, neurologic deficits; DM, distant (extracerebral) metastases; ECOG-PC, eastern cooperative oncology group - performance status; epi, epileptic seazure; F, female; FTC, follicular thyroid cancer; GBq, giga becquerel; left/right/both hemispheres; M, male; MTC, medullary thyroid cancer; N, number; NA, not applicable; OP, surgical resection; PTC, papillary thyroid cancer; PDTC, poorly-differentiated thyroid cancer; pres, intreased intracerebral pressure; RAIT, radioactive-iodine therapy; SRS, stereotactic radiotherapy; TC, thyroid cancer, TNM, tumor, nodes, metastases; WBRT, whole brain radio therapy; 2 L, second line*

### Clinical characteristics before BM diagnosis

The primary therapeutic strategy differed between the histological subtypes. Overall, 15/18 patients with DTC had received RAI therapy during the course of the disease. The median number of RAI treatments was 2.5 (range 0–10), with a median dose of 101.45 mCi (range101-1,487 mCi-) being administered. Six patients received a dose of > 600 mCi, however, information about the received RAI dose was only available for 11/20 patients. The patient with MTC had received chemotherapy (substance unknown) after thyroidectomy and the patient with ATC received two cycles of doxorubicin before diagnosis of BM.

In total, 8/20 (40%) patients had organ metastases at TC diagnosis; two had both pulmonary and osseous metastases and 6 had pulmonary, osseous and BM. Hence, all patients, who presented with distant metastases at primary diagnosis, had at least two sites of distant metastases (lung, bones and/or brain) and none presented with BM as the only metastatic disease. At diagnosis of BM, all patients had pulmonary metastases. Interestingly no patient had a singular pulmonary lesion but 18/20 patients had several (at least three) lesions located in one lung and two patients had bilateral, disseminated pulmonary lesions. Ten patients had bone metastases including eight patients with a singular bone lesion located in a vertebral body, rib, scapula, tibia, femur, or pelvis and two patients with disseminated bone metastases. For more details regarding baseline patient characteristics, see Table 1.

In addition to the 8 patients with extracranial distant metastases at initial diagnosis, 12 patients developed distant metastases during course of disease. The median time to development of distant metastases from initial diagnosis of TC was 8.7 years for DTC with a wide range between 1.5 and 42 years (both patients had FTC). Patients with FTC developed distant metastases earlier compared to patients with PTC (1.8 vs. 9.5 years, respectively, p = 0.57). 6 patients with FTC and 5 patients with PTC had localized disease at primary diagnosis. The patient with MTC developed distant metastases 19 years after initial diagnosis and the patient with ATC presented with distant metastases at primary diagnosis. As expected, at diagnosis of BM, distant metastases were predominately located in the lung and bones among 19/20 patients (95%) and one patient had distant metastases in the lung, bones and adrenal glands.

### Clinical characteristics at BM diagnosis

In total, 6/20 patients had BM at TC diagnosis. Of those, two patients had PTC, three had FTC and one had ATC. No patient presented with BM as the only metastatic site, as all patients also had synchronous distant metastases in the lung and bones. The median time from TC diagnosis to BM was 8.5 years for 13 DTC patients (range 0.7–41.2 years). Patients with FTC (n = 6) developed BM earlier compared to patients with PTC (n = 5) with 4 vs. 13 years, respectively (p = 0.74). The median age at diagnosis of BM was 68 years (range 35–75 years). Patients with FTC developed BM at a numerically slightly younger age compared to patients with PTC (median age at diagnosis of BM: 62 vs. 72 years, respectively). The patient with MTC was 71 and the patient with ATC 66 years old at diagnosis of BM. The ECOG-PS was worse at diagnosis of BM compared to the ECOG-PS at diagnosis of TC. Two out of 20 patients (10%) had an ECOG-PS of 0 (compared to 12 patients at diagnosis of TC), 16 (80%) patients had an ECOG-PS of 1 (compared to 8 patients at diagnosis of TC) and two (10%) patients had an ECOG-PS of 2.

### Neurological symptom burden, BM characteristics and treatment

Notably, almost all BM were symptomatic at initial manifestation (19/20, 95%). In detail, 11 patients presented with neurological deficits, five had symptoms of increased intracranial pressure and three had epileptic seizures. Only one patient with FTC had asymptomatic BM diagnosed coincidentally on a routine screening after RAI therapy. Regarding BM pattern, 13/20 patients had singular BM predominately located in the left hemisphere (8/13), three BM were located in the right hemisphere and two in the cerebellum. Two patients had two BM, in both patients located in the left and in the right hemisphere, 3/20 patients had three BM and two patients had more than three BM. Information about the size of BM was available for 13/20 patients. The size of BM ranged from 0.3 to 7 cm (patient with FTC). Seven patients had BM with a diameter of < 2 cm. The others had BM sizes between 3 and 7 cm. See Table 1 for a detailed list of BM-characteristics.

The most common upfront treatment for BM was total resection (n = 7), followed by stereotactic radiosurgery (SRS, n = 4), resection and SRS (n = 4), whole brain radiotherapy (WBRT, n = 3) and best supportive care (BSC, n = 2). If only looking at the DTC cohort, this distributed as follows: among the PTC patients, two patients had a resection, two a SRS, three a SRS and resection and one a WBRT; among the FTC patients, five patients had a resection, one a SRS, one a SRS and resection, one a WBRT and two patients with FTC were treated with BSC. Regarding the four patients with combined therapy, one patient with a singular BM was surgically resected and the resection area was subjected to subsequent SRS and three patients had multiple lesions, of which the biggest were surgically removed and the smaller BMs were treated with SRS. In total, 8/20 patients had a second BM treatment (SRS n = 5, resection n = 1, and WBRT n = 2). Only three patients had a third BM specific treatment (surgery, SRS and WBRT). No DTC patient received RAI for treatment of active BM.

Seven patients received additional systemic therapies. Of those, three patients were treated with chemotherapy for metastatic TC (two DTC, one ATC) before 2007 (i.e., before approval of TKIs). The patient with ATC received doxorubicin. The CHT regimen for the DTC patients remained unknown. Three patients with BM from DTC diagnosed after 2017 were treated with lenvatinib after progression upon RAI therapy. Lenvatinib was started before diagnosis of BM for one and after diagnosis of BM for two patients. No objective intracranial response was observed but patients remained stable for 2, 4 and 5 years respectively and two of these patients were still alive at time of analysis after a median follow up time of 75 months. Finally, one patient with FTC received temozolomide. This approach, however, was not successful as the therapy had to be discontinued after one cycle due to severe side effects (hematologic toxicities). See Table 1 for detailed clinical characteristics, treatment and outcome of patients with BM and TC.

### Outcome after BM diagnosis

The overall PFS time after the first BM-specific treatment was two months for patients with PTC, 7 months for patients with FTC, 139 months for the MTC and only 2.6 months for the ATC patient (p = 0.266).

**Fig. 1:**
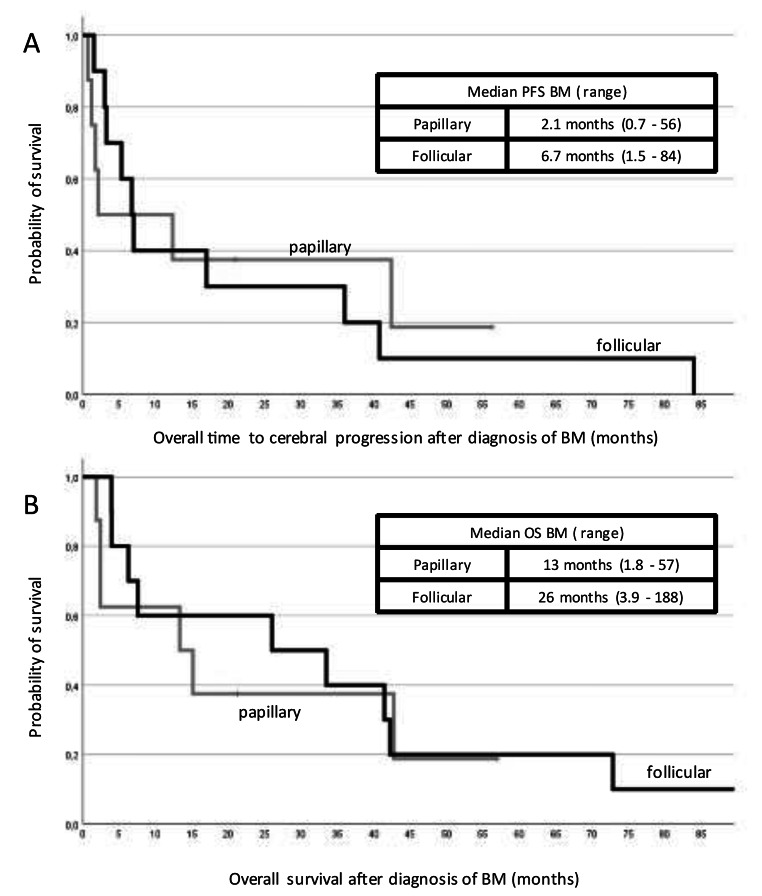
PSF and OS since BM diagnosis Progression free survival (**A**) and median overall survival (**B**) since diagnosis of brain metastases for papillary (n = 8) and follicular (n = 10) thyroid cancer.

The median survival after diagnosis of DTC was 6.5 years (range 0.2–42 years) and the median survival after diagnosis of BM was 15 months (range 1.8–188 months, see Fig. 1). The patient with MTC lived 33 years after diagnosis of TC and 12 years after diagnosis of BM. The patient with ATC lived nine months after diagnosis of TC and 2.6 months after diagnosis of BM. Patients with FTC had a numerically shorter OS from BM compared to patients with PTC (13 vs. 26 months, p = 0.309). Ten patients died due to intracranial progression. Patients undergoing surgery had a better long-term outcome with a longer median OS compared to patients who didn’t have a surgical resection, however, this was not statistically significant (4 vs. 33 months, p = 0.406). This was also observed in the DTC only cohort (4 vs. 42 months, p = 0.11). Patients with singular brain lesions (n = 12) had a trend for longer survival than patients with more than one brain lesion (26 vs. 13 months, p = 0.22).

## Discussion

According to the literature, the development of BM constitutes a rare event in TC patients, documented in approximately 1% of DTC, 4% of MTC and 10% of ATC patients. In view of the rarity of BM in TC and the lack of systematic data regarding management and clinical outcome, we have retrospectively reviewed the Vienna Brain Metastases Registry to investigate BM distribution, neurological symptom burden and BM-directed therapies in this highly distinct cohort of patients.

Our findings again underscore the exceedingly rare occurrence of BM from TC, with only 20/6047 patients (0.3%) in the Vienna Brain Metastases Registry having an underlying diagnosis of TC. The large majority was related to DTC (18/20, 90%) with only one patient each presenting with MTC and ATC. The predominance of DTC is in keeping with epidemiological data but analysis of subtypes of DTC requires further notice. In our cohort, 10/18 DTC patients (55%) had FTC, underscoring the more aggressive nature of FTC which only accounts for roughly 20% of all TC cases. Data in the literature regarding this observation, however, are conflicting: Shaha et al. reported that distant metastases including BM occurred more often in FTC (22%) compared to 10% in PTC, while other series reported BM to be more common in PTC [30–35]. Of clinical relevance is the fact that we also observed an earlier development of BM in FTC compared to the PTC cohort (2 vs. 10 years, respectively), suggesting that one should probably be more alert in these patients if neurological symptoms occur. Interestingly, no patient with oncocytic or poorly differentiated carcinoma was documented in our database, which is in contrast to other publications [4, 8, 9, 20–26]. See Table 2 for an overview of comparable patient cohorts reported in the literature.

**Table 3:** Publications about brain metastases from thyroid cancer since 2010

Author	Year	No. of patients	Median time to BM in months (range)	Median time from BM to death in months (range)	Study population
**Current study**	**2022**	**20**	**36 (0–495)**	**15 (2–188)**	**8 PTC, 10 FTC, 1 MTC, 1 ATC**
Bunevicius et al. [20]	2021	42	5 (0–49)	14 (3–58)	35 PTC, 7 FTC
Kim et al. [21]	2021	35	102 (NA)	NA	25 PTC, 1 FTC, 1 MTC, 8 ATC
Wu et al. [8]	2021	22	NA	18 (1–60)	14 PTC, 6 FTC, 2 unknown histology
Slutzky et al. [22]	2018	10	40 (9–207)	15 (2–59)	3 PTC, 3 FTC, 2 PDTC, 1 tall cell, 1 insular
Gomes-Lima et al. [44]	2018	24	11 (0–37)	19 (0–119)	15 PTC, 7 FTC, 1 PDTC, 1 unknown
Hong et al. [4]	2018	16	76 (1–216)	27 (NA)	7 PTC, 4 FTC, 1 Hurthle, 2 PDTC, 1 MTC, 1 ATC
Choi et al. [9]	2016	37	46 (0–368)	9 (1–109)	32 PTC, 3 FTC, 2 PDTC
Saito et al. [23]	2016	25	73 (0–182)	42 (0–83)	18 PTC, 7 FTC
Lee et al. [24]	2015	10	51 (0–150)	33 (1–78)	6 PTC, 3 FTC, 1 PDTS
Henriques et al. [25]	2014	21	NA	7 (NA)	12 PTC, 5 FTC, 4 PDTC
Bernad et al. [26]	2010	12	42 (0–516)	21 (NA)	9 PTC, 2 Hurthle, 1 MTC

*ATC, anaplastic thyroid cancer; BM, brain metastases; FTC, follicular thyroid cancer, MTC, medullary thyroid cancer; NA, not applicable; No, number; PTC, papillary thyroid cancer; PDTC, poorly-differentiated thyroid cancer*

The observed time from diagnosis of TC to BM was wide with a range from synchronous presence to up to 42 years from diagnosis of TC, again underscoring the different prognosis depending on the histological TC subtype. The median time to BM diagnosis was 36 months in the entire cohort with 6 patients presenting with synchronous BM and 102 months in patients who without BM at TC diagnosis. Interestingly, we observed BM only in patients with additional distant metastases, but not as an isolated event. Current guidelines do not support routine screening for BM in TC patients, and our data also do not suggest screening for BM in asymptomatic patients. However, in the subgroup of FTC and/or patients with distant metastases and neurological symptoms the treating physicians should be alert of the possibility of BM. This is further supported by the fact that all but one patient presented with neurological symptoms leading to diagnosis of BM (n = 11 with neurological deficits, 5 with increased intracranial pressure, 3 with seizures). Most patients presented with singular lesions (13/20), which were most commonly located in the left hemisphere (8/13). Finally, one could also consider brain imaging in patients with elevated tumor markers and absence of radiological extracranial lesions or progression, given the high sensitivity and specificity of biomarkers in DTC and MTC.

In general, local therapies appear to effectively control BM in the majority of patients judging from the scarce data in the literature [33]. Some authors reported a statistically significant longer BM-specific survival among patients who underwent resection compared to patients who did not [4, 8, 9, 30, 33]. In keeping with this, our patients treated with surgery had a better long-term outcome compared to patients who could not undergo resection (median OS from BM: 33 vs. 4 months). This also applied to the specific cohort of DTC patients (4 vs. 42 months, p = 0.11). Even though the difference was not statistically significant in our cohort, a significant difference was reported by other authors [1–5]. This again supports open surgery as the treatment of choice for BM, even in patients with impaired general condition [8]. While SRS could also be shown as effective in our series in line with literature reports [20, 32], we and several authors found resection to be an independent covariate for prolonged survival [4, 30, 33, 34, 36]. In analogy to other series, our analysis does not allow for estimation of RAI therapy as a therapeutic option for BM, which has only been anecdotally reported in the literature [35].

TKIs are increasingly being used in patients with radio-iodine refractory TC but little is known about their intracranial efficacy. To date, several multi-TKIs are approved for DTC and MTC. Effects for controlling BMs and improvement of QOL have only occasionally been reported [37, 38]. The potential risk of intracranial bleeding in BM treated with multi-TKIs implicating anti-angiogenic effects needs to be considered but appears limited based on available data [38]. Also, our data do not allow for additional extrapolation on the value of TKIs in patients with BM, as only three patients received a TKI (lenvatinib) in our cohort. Lenvatinib was started before diagnosis of BM in one and after diagnosis of BM in two patients. Whereas no objective intracranial response was observed, two of three patients treated with lenvatinib experienced improved QoL and were still alive at time of analysis.

Relatively promising are data for intracranial activity of recently approved novel driver mutation based targeted therapies. Selective RET-inhibitors such as selpercatinib and pralsetinib are effective therapeutic options for *RET* -altered tumors, and both compounds have shown considerable intracranial efficacy of 91% in *RET* fusion-positive NSCLC in the pivotal studies [39, 40]. *RET* alterations occur in over 60% of sporadic MTC, 98% of hereditary MTC and 10–20% of PTC [41], thus rendering these compounds of interest in selected patients with TC and BM. In addition, the NTRK inhibitors larotrectinib and entrectinib are approved for *NTRK* fusion positive DTC and MTC and particularly entrectinib is characterized by a high central nervous system penetration. An intracranial response rate of 64% was reported for entrectinib in the combined ALKA/ STARTRK trial [42]. While *NTRK* fusions are extremely rare in TC (< 2%), these data still support NGS testing at least following standard therapy. Furthermore, *BRAF V600E* mutations are a common finding in PTC and ATC associated with a more aggressive course. *BRAF V600E* can be targeted by BRAF +/- MEK inhibitors which show relevant intracranial control with most data again deriving from NSCLC. However, *BRAF*-targeted therapy is currently only FDA approved in ATC and in our cohort, only two PTC patients had a *BRAF V600E* mutation. Both had a short OS from diagnosis of TC of only 3 and 26 months, respectively. No patients in our cohort were treated with checkpoint inhibitors (CPI), and data regarding TC and CPIs remain scarce. First cohorts such as in the ATLEP study (lenvatinib and pembrolizumab for ATC) are being defined and based on experience of other tumor entities intracranial control is expected for CPI therapy also in TC [43].

Regarding OS, the median OS in our series after diagnosis of BM was 15 months (range 1.8–188 months) for patients with DTC, with a median OS of 6 years after initial diagnosis of TC (range 0.3–42 years). Again, FTC patients had a shorter OS from BM compared to the PTC cohort (13 vs. 26 months). These figures appear to be roughly in line with other series, with a median OS from diagnosis of BM reported to be between 7 and 33 months, depending on histological subtype, patient’s performance status, the time from TC to BM diagnosis, number of BM sites and BM specific treatment [9, 20, 23, 26, 32].

The retrospective nature, the small sample size of only 20 patients with mixed histologies (while being in the medium range compared to other single center cohorts, see Table 1), as well as the absence of systematic assessment of further pathological characteristics such as *BRAF/RET* mutations or *NTRK* fusions are caveats of our analysis. Furthermore, due to the BM-specific focus of the underlying registry, these data do not allow extrapolation of prognostic and predictive factors rendering TC patients more prone to developing BM or regarding the general incidence of BM in TC patients.

Our analysis nevertheless provides a real-world analysis of a rare patient cohort treated at a tertiary referral center. We confirm that BM-development is associated with the histological subtype of TC and cautiously hypothesize that, in contrast to ongoing guidelines, screening for BM might be indicated for selected patients at individual intervals. This is supported by the finding that many patients present with symptomatic singular lesions, suggesting the possibility of improved local treatment options if detected early enough. This is underscored by the fact that aggressive (local) BM-specific treatment, preferable neurosurgery, appears to provide the best control for TC BM. In addition, we observed BM only in patients with additional distant metastases, which might be a further indicator for patient selection and screening. Finally, following recent data on personalized medicine approaches, we encourage NGS testing in all patients with advanced disease.

In conclusion, BM constitute a generally poor prognostic factor in TC patients, but individual patients experience long-term survival following local therapy. More information about clinical, pathological and molecular risk factors is needed to allow provision of distinct guidelines for the management of BM in TC. For the time being, patients should be managed on an individual basis in a tertiary referral center with access to (neuro-) surgery.

## Data Availability

Enquiries for further data can be directed to the corresponding author.
